# Visualization of comparative genomic analyses by BLAST score ratio

**DOI:** 10.1186/1471-2105-6-2

**Published:** 2005-01-05

**Authors:** David A Rasko, Garry SA Myers, Jacques Ravel

**Affiliations:** 1 The Institute for Genomic Research, 9712 Medical Center Drive, Rockville, MD 20850 USA

## Abstract

**Background:**

The first microbial genome sequence, *Haemophilus influenzae*, was published in 1995. Since then, more than 400 microbial genome sequences have been completed or commenced. This massive influx of data provides the opportunity to obtain biological insights through comparative genomics. However few tools are available for this scale of comparative analysis.

**Results:**

The BLAST Score Ratio (BSR) approach, implemented in a Perl script, classifies all putative peptides within three genomes using a measure of similarity based on the ratio of BLAST scores. The output of the BSR analysis enables global visualization of the degree of proteome similarity between all three genomes. Additional output enables the genomic synteny (conserved gene order) between each genome pair to be assessed. Furthermore, we extend this synteny analysis by overlaying BSR data as a color dimension, enabling visualization of the degree of similarity of the peptides being compared.

**Conclusions:**

Combining the degree of similarity, synteny and annotation will allow rapid identification of conserved genomic regions as well as a number of common genomic rearrangements such as insertions, deletions and inversions. The script and example visualizations are available at: .

## Background

In the decade since the publication of the *Haemophilus influenzae *genome sequence in 1995 [1], 191 microbial genomes have been completed, with another 276 in progress [2]; as of October 14, 2004). Multiple strains of the same organism, or multiple species of the same genus are being sequenced or have been completed, making comparative genomic analysis possible on an unprecedented scale. As the technology continues to improve, the number of completed microbial genome sequences will further increase – a major challenge of the comparative genomic era is to fully exploit this data. However, the development of tools for analysis of such data sets has not kept pace.

BLAST analysis has become a ubiquitous method of interrogating new sequence data, but there are limitations to using BLAST alone as a discriminating tool. Many other methods and individuals use BLAST output E-values as a criterion for data parsing. While this measure may be efficient, the output is often skewed by both the database used for comparison and the length of the match [3]. Small regions of high similarity can generate an artificially low E-value and negate the global level of similarity exhibited by the sequence. This bias is eliminated when using the BLAST raw score as it is directly derived from the similarity of the match. However the value of the BLAST score varies with the length of the peptide queried, and hence is not suitable alone for comparative analysis using universal cutoffs [4].

Several other tools utilize the BLAST algorithms to compare nucleotide or peptide sequences from genome projects. The Wellcome Trust Sanger Institute ACT software [5] can display nucleotide similarity between two genomes based on BLASTN E-value. ACT builds upon Artemis and displays regions of high similarity mapped on the genome annotation. The GenomeComp tool [6] displays a similar analysis also based on BLASTN E-values to compare genome sequences. NCBI Taxplot, a three-way genome comparison tool based on precomputed protein BLASTP E-values displays a point for each protein in the Reference genome based on the best alignment with proteins in each of the two genomes being compared [7]. On the other hand, the SimiTri program utilizes BLASTP comparisons of three proteomes and uses the raw BLAST score, not E-values. However, only protein similarity data is represented and no information on the comparative structure of the genomes is provided [8]. Moreover, the SimiTri program does not address BLAST artifacts derived from the size of the database or the length of the match. This paper describes the BLAST score ratio (BSR) algorithm that enables comparative analysis of multiple proteomes, together with visualization of genome structure (synteny).

BSR analysis is a departure from traditional genome scale analyses as it overcomes the limitations of BLAST E-values in comparative studies by normalizing the BLAST raw scores. BSR analysis is a tool for the rapid comparison of complete proteomes of any three genomes, and enables a visual evaluation of the overall degree of similarity of these proteomes and their genomic structure.

## Implementation

We have implemented the BSR algorithm using Perl. The inputs are the predicted proteomes of each of the three genomes under analysis, formatted as multi-FASTA files. An additional file for each of the proteomes is required. This file must contain a unique identifier, matching the FASTA header of the corresponding peptide in the multi-FASTA file, the relative genomic location of the start and stop of the coding region as well as the annotation for each peptide. The user selects one proteome as the "Reference"; the other two proteomes are termed "Query_1_" and "Query_2_" respectively. Initiation of the script results in each of the putative peptides in the Reference proteome being compared to all of the other peptides in the Reference and Query proteomes using NCBI BLASTP.

**Figure 1:**
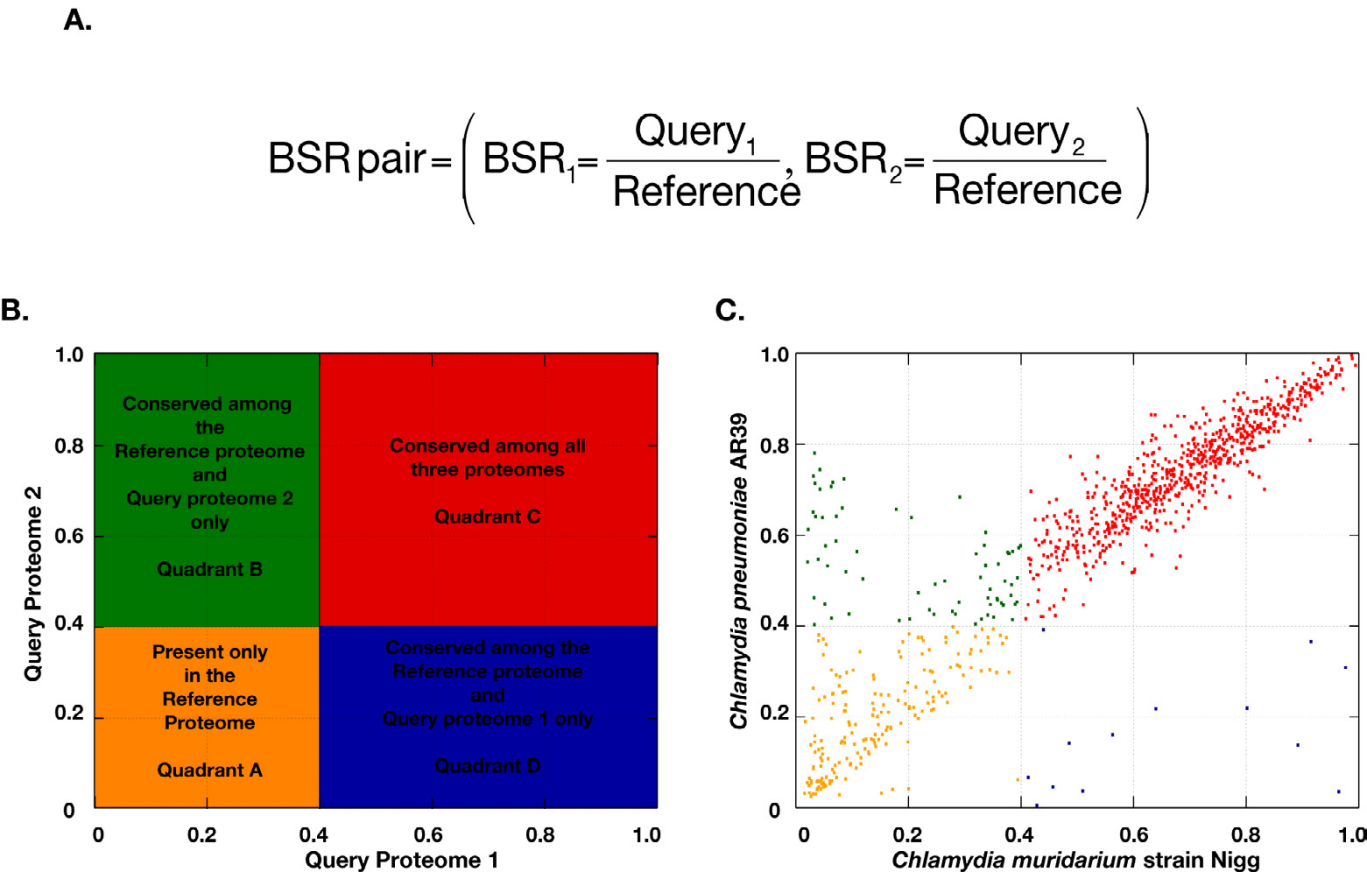
**BSR rationale and scatter plot example. A. **BLAST score ratio analysis (BSR) calculation demonstrating how the two coordinates for plotting in figures B and C are calculated. **B. **Locations of the peptide spot revels the similarity that the peptide has to the two Query genomes. Use of a 0.4 separator is based on ~30% amino acid identity over 30% of the length of the peptide [10]. **C. **Sample data obtained from comparison of *Chlaymidia caviae *GPIC (GenBank Accession Number AE015925) to the proteomes of *Chlamydia muridarum *strain Nigg (GenBank Accession Number AE002160) and *Chlamydia pneumoniae *AR39 (GenBank Accession Number AE002161) [17]. Each point in the figure represents a single peptide in *Chlaymidia caviae *GPIC This analysis reveals that while these organisms are very similar, *C. caviae *is more similar to *C. pneumoniae *AR39 than *C. muridarum *strain Nigg due the skew of peptides with a slope of greater than 1.

The BSR is then computed as follows. The BLAST raw score for each Reference peptide against itself is stored as the Reference score. Each Reference peptide is then compared to each peptide in the Query_1 _and Query_2 _proteomes with each best BLAST raw score recorded as Query_1 _and Query_2_, respectively (Figure 1). The BSR is calculated by dividing the Query score by the Reference score for each Reference peptide (Figure 1A). Thus, for each peptide in the Reference genome, two numbers are generated, one from each from the best matches in Query_1 _and Query_2_, thus normalizing all scores in the range of 0 to 1. A score of 1 indicates a perfect match of the Reference peptide to a Query peptide and score of 0 indicates no BLAST match of the Reference peptide in the Query proteome. The BLAST raw score is used rather than the E-value for the BLASTP results as it more accurately accounts for the length of the similarity between the Reference and Query peptides [4,9]. This normalized pair of numbers can be plotted as coordinates in Cartesian space for each peptide in the Reference proteome, enabling the visualization of the entire Reference proteome in comparison to the two Query proteomes (Figure 1B).

## Outputs

Following calculation of the BSRs, a number of output files are generated, including both text and graphical formats. The text files are tab-delimited for ease of parsing; filenames are derived from the named proteome files used as input into the script. The R_Q1_Q2.txt (Reference_Query_1__Query_2_.txt) output contains an ordered list including the Reference peptide unique identifier, annotation, and Reference BLAST raw score, in addition to the unique identifier of the best hits in the Query proteomes, corresponding BLAST raw scores and the calculated BSR. Additionally, four unique files are generated corresponding to the peptides within the four quadrants delineated in Figure 1B. The four quadrants are derived from a BSR threshold value of 0.4, which was empirically determined to represent approximately 30% amino acid identity over approximately 30% of the peptide length, a commonly used threshold for peptide similarity [10]. This threshold value can be adjusted using the "-C" option (see help file).

The graphical output files are viewed with Gnuplot [11] to reveal the global similarity of the compared genomes as well as the level of conserved genome structure. PostScript and xfig [12] graphic files are subsequently generated by Gnuplot. The scatter or similarity plot provides an overall view of the level and number of similar and dissimilar proteins in the Reference proteome when compared to the Query proteomes (Figure 1C). The regions of the graph are color-coded depending on the level of similarity between the three genomes (Figure 1B). Quadrant A (BSR < 0.4), colored in orange, contains peptides unique to the Reference proteome with little similarity in either of the Query proteomes. Quadrant C (BSR > 0.4), colored Red, contains peptides that have significant similarity in all three compared proteomes. Quadrant B, colored green, contains Reference peptides with similarity to only Query proteome 2, whereas Quadrant D, colored blue, contains Reference peptides that have similarity to only Query proteome 1.

**Figure 2:**
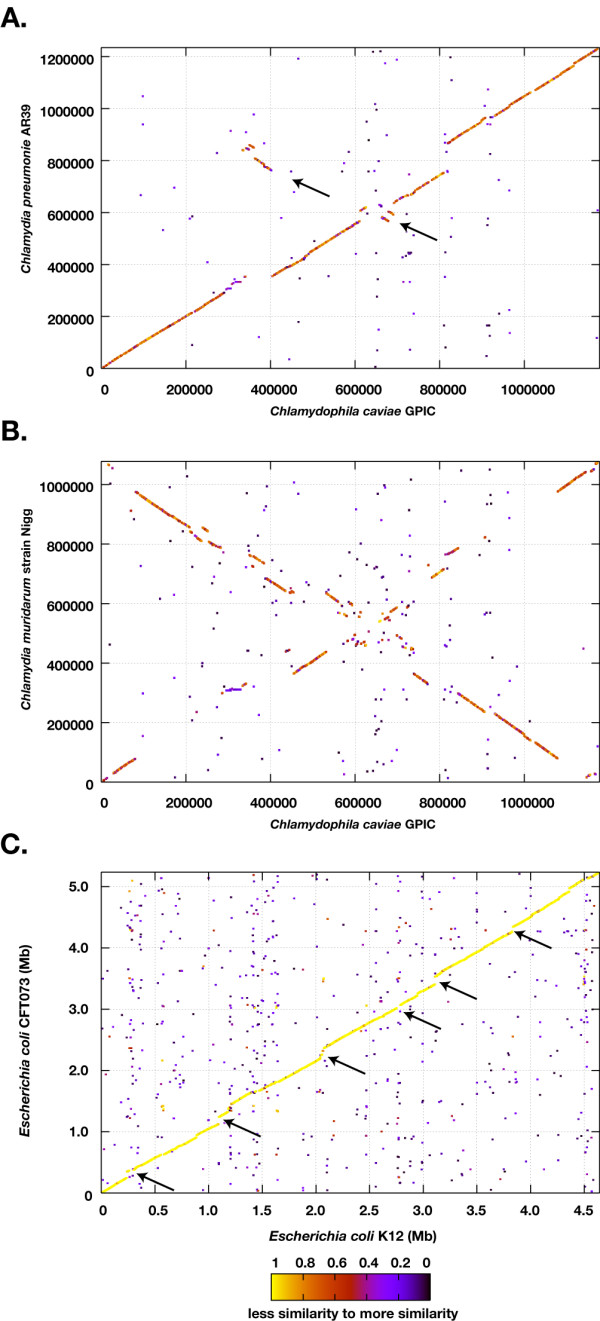
**Genome structure visualization. **Direct comparison of two genomes at a time demonstrating some examples of large-scale genomic rearrangements. Each protein is plotted by the genomic location of the coding region and is color-coded by the degree of similarity based on the BSR as is demonstrated in the legend. **A. **Comparison of *C. caviae *GPIC and *C. pneumoniae *AR39. This comparison contains two genomic rearrangements of different sizes as indicated by the arrows. **B. ***C. caviae *GPIC and *C. muridarum *strain Nigg comparison reveals a more extensive genomic rearrangements suggesting that while proteomically these organisms are similar the genomes have diverged significantly. **C. ***E. coli *CFT073 (GenBank Accession Number AE014075) vs. *E. coli *K12 (GenBank Accession Number U00096). *E. coli *CFT073 contains a number of unique insertions that are represented as breakpoints in the plot and highlighted with arrows. The high level of synteny and similarity are exhibited by these genomes.

Two additional plots, termed synteny plots, are generated, one for comparison of the Reference proteome to each Query proteome, by plotting the genomic location of the Reference peptide on the X-axis and the genomic location of the most similar Query peptide on the Y-axis. This plot alone would demonstrate the level of synteny (conservation of gene order) between the two genomes [13], however, an additional level of information is included by coloring each point based on the BSR (see legend Figure 2). The color provides an additional visual clue to the global level of similarities of the proteomes. For example genomes can be highly syntenic with relatively low levels of proteomic similarity as is shown in Figure 2A and 2B or they may have a high degree of protein similarity **and**conserved genome structure (Figure 2C).

**Figure 3:**
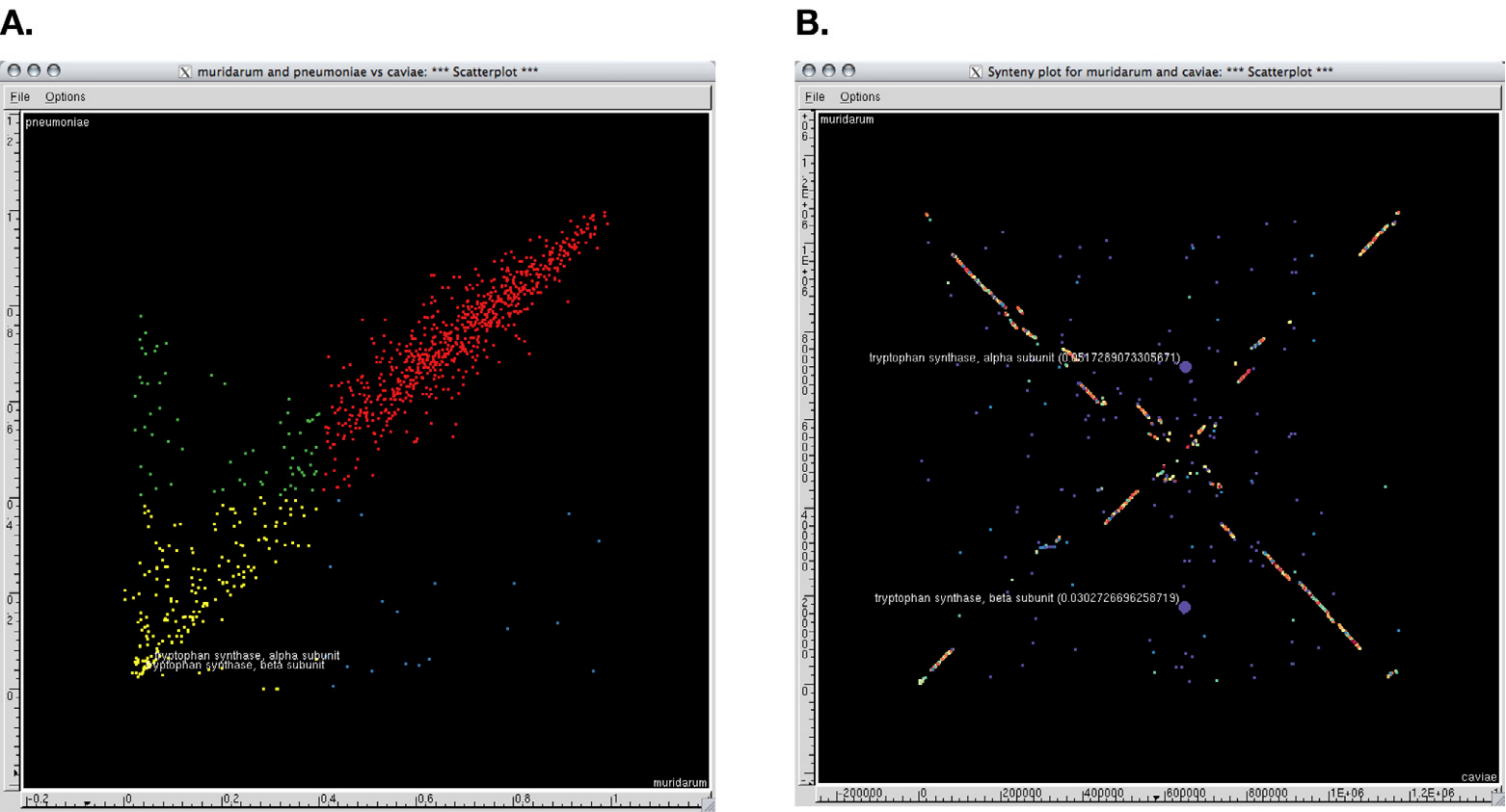
**Visualization with GGobi. **GGobi screenshots of the graphical outputs from the BSR. The proteins for tryptophan synthase alpha and beta subunits are highlighted as they were unique in the *C. caviae *genome and represented a significant metabolic adaptation of this species in comparison to the other species compared [17]. **A. **The scatter plot represents the same figure as shown in Figure 1C, however the interactive nature of GGobi allows visualization of the annotation associated with any of the peptides. **B. **Synteny plots as seen in GGobi. These same genes from Figure 3A can be highlighted in the in the synteny plots and the genomic location can be observed. To take advantage of the usefulness of the interactive mouseover the BSR is included with the annotation.

The Gnuplot, PostScript and xfig outputs allow publication-quality, global visualization of the similarity and synteny of the selected genomes. However these formats do not allow the annotation associated with individual peptides to be viewed interactively. To overcome this limitation, additional XML files for the similarity and synteny plots described above are generated. These files are the input for the freely available GGobi software. GGobi is a data visualization system for viewing high-dimensional data [14]. The tools provided in the GGobi software package allow the annotation associated with individual points within the similarity and synteny plots to be viewed interactively (Figure 3).

The GGobi package also allows the expansion of the BSR approach to include more than three genomes or other additional parameters associated with proteomic or genomic data, enabling interactive, user-driven exploration of these complex datasets. The current BSR implementation uses three genomes as input; however, additional genomes can readily be added as new dimensions simply by repeating the analysis with the same Reference genome and varying the Query genomes. Additional non-BSR dimensions are readily included, such as pI or %GC, or factors such as surface localization or some other feature of the peptides of interest.

## Results

Genome structure is often altered during the evolution of species [13]. Visualization of this structure often lends insight into genome evolution and examination of the various BSR outputs rapidly reveal alterations of the genome structure as well as the overall similarity of the two Query proteomes to the Reference proteome. The genomes of the Order Chlamydiales (Figures 1, 2 A and 2B) provide an example of this insight. In Figure 1a large proportion of the peptides are conserved, with 71.7% of the proteins shared between all three proteomes. If the Query proteomes are further used as the Reference proteome and vice versa we still see a similar trend (data not shown). Additionally, the proteome of *C. pneumoniae *AR39 (GenBank Accession Number AE002161) is more similar to *C. caviae *GPIC (GenBank Accession Number AE015925) than *C. muridarum *strain Nigg (GenBank Accession Number AE002160) as 7.3 % of the proteome is shared between only *C. caviae *GPIC and *C. pneumoniae *AR39 compared to only 1.6% between *C. caviae *GPIC and *C. muridarum *strain Nigg. Finally, Figure 1 demonstrates that 19.4% of the *C. caviae *proteome has no significant hit to any of the peptides in the Query proteomes, although many of these peptides (78.2%) are currently annotated as hypothetical.

From the analysis in Figure 1 we could conclude that the chlamydial proteomes are extremely similar and suggest that the genome structure will also be similar. However, the synteny plots in Figure 2A and 2B demonstrate that while the chlamydial proteomes exhibit a high degree of similarity, there is significant alteration in the genomic structure. The comparison of the proteomically similar organisms, *C. caviae *GPIC and *C. pneumoniae *AR39 reveals that the genomes contain two points of inversion (arrows in Figure 2A). One of these points of inversion is centered on the terminus of replication. There are more extensive genomic rearrangements between the *C. caviae *GPIC and *C. muridarum *strain Nigg genomes (Figure 2B). The additional color information extends the utility of these synteny plots. While the chlamydial genomes show regions of conserved synteny, as demonstrated by the peptides in the same genomic location forming a line with a slope of 1 or -1, the absolute degree of similarity between the peptides, demonstrated by color indicates divergence. By contrast the synteny plot of two *Escherichia coli *genomes (Figure 2C) demonstrates a high level of synteny with a number of unique insertions, however no inversions are present. Moreover the color dimension on this plot reveals that unlike the chlamydial proteome comparisons the *E. coli *proteomes have a high level of similarity **and**synteny.

In the analysis of the Chlamydial proteomes using BSR score and BLAST E-values approximately 1% of peptides examined have a BSR score > 0.4 and BLAST E-value > 1 × 10^-15^. These peptides were all very small in size (< 70 amino acids) and greater than 50% amino acid identity. This group of peptides is more readily identified by BSR analysis than BLAST E-value, which is artificially low due to the small peptide size. Additionally, peptides that have a BSR score < 0.4 but a BLAST E-value < 1 × 10^-15 ^correspond 7.8% of the proteome. These represent divergent peptides with an artificially high BLAST E-value score resulting from limited regions of identity. The BSR analysis more accurately classifies these peptides based on the amino acid identity over the entire peptide. As the BSR comparison utilizes a single genome as a reference, the BSR score is calculated using a unidirectional best BLAST hit. However, when the Chlamydial proteomes were compared only one case in over 1000 could be found with a BSR score > 0.4 that was not also a bidirectional best BLAST hit.

## Conclusions

The BSR approach allows rapid evaluation of the level of conservation of any three proteomes and the degree to which the genome structure between the three genomes is similar. While in this report we discuss the applications of this approach to whole genomes, the analysis has been performed on portions of genomes such as genomic or pathogenicity islands, plasmids and phage to identify peptide similarity and regional structure.

More genome sequences are being generated from closely related organisms – a trend which shows no sign of abating. The BSR approach has become a crucial tool in our comparative genomics armamentarium and has been utilized in a number of genomic comparisons, revealing regions of similarity and difference between both closely and distantly related organisms [10,15,16].

## Availability and requirements

**Project name: **BSR.pl

**Project homepage: **

**Operating System: **Unix and MacOS X

**Programming language: **Perl

**Other requirements: **Perl Statistics::Descriptive module 

**License: **None

**Any restrictions to use by non-academics: **None

## List of abbreviations

BSR – BLAST score ratio; BLAST – basic local alignment search tool.

## Authors' contributions

DAR, GSAM and JR conceived and implemented the first versions of BSR and prepared the manuscript. All authors have read and approved the final manuscript.
